# Reduced Ropivacaine Volume with Perineural Dexamethasone in PENG Block for Total Hip Arthroplasty: A Randomized Controlled Trial

**DOI:** 10.3390/jcm14248722

**Published:** 2025-12-09

**Authors:** Tomasz Reysner, Agnieszka Neumann-Podczaska, Pawel Pietraszek, Aleksander Mularski, Grzegorz Kowalski, Przemyslaw Daroszewski, Malgorzata Reysner

**Affiliations:** 1 Department of Palliative Medicine, Poznan University of Medical Sciences, Os. Rusa 55, 61-245 Poznań, Poland; 2 Senior Institute, Department of Medical and Health Sciences, Vizja University, 01-043 Warsaw, Poland; 3 Department of Anesthesiology and Intensive Care, Poznan University of Medical Sciences, 61-245 Poznań, Poland; 4 Department of Forensic Medicine, Institute of Medical Sciences Collegium Medicum, University of Zielona Góra, 65-516 Zielona Góra, Poland; 5 Department of Organization and Management in Health Care, Poznan University of Medical Sciences, 61-245 Poznań, Poland

**Keywords:** pericapsular nerve group block (PENG), total hip arthroplasty, ropivacaine, perineural dexamethasone, low-volume local anesthetic, motor-sparing regional anesthesia, opioid-sparing analgesia, quadriceps strength, elderly patients, randomized controlled trial

## Abstract

**Background/Objectives**: The pericapsular nerve group (PENG) block is increasingly used as part of multimodal analgesia for total hip arthroplasty (THA). However, standard high-volume local anesthetic regimens may impair motor function. The addition of perineural dexamethasone could allow for volume reduction while maintaining analgesic efficacy and minimizing quadriceps weakness. This study evaluated whether adding dexamethasone to a low-volume PENG block prolongs analgesia, reduces opioid consumption, maintains pain control, and preserves motor function compared to a standard-volume PENG block. **Materials and Methods**: In this randomized controlled trial (NCT06470334), 60 adult patients undergoing THA via the direct superior approach received either a standard-volume PENG block with 20 mL of 0.2% ropivacaine (PENG group) or a low-volume block with 10 mL of 0.2% ropivacaine plus 4 mg of perineural dexamethasone (PENG + DEX group). The primary outcome was time to first rescue opioid. Secondary outcomes included total 48-h opioid consumption (oral morphine equivalents), pain scores (numeric rating scale, NRS) at rest and during movement, and quadriceps muscle strength at predefined postoperative intervals. **Results**: The PENG + DEX group demonstrated a significantly longer time to first opioid administration (15.0 ± 1.5 h vs. 9.1 ± 1.7 h; *p* < 0.0001) and reduced total opioid consumption within 48 h (2.3 ± 3.1 mEQ vs. 5.0 ± 4.4 mEQ; *p* = 0.0120). Pain scores were similar at 4 h but significantly lower in the PENG + DEX group at 8, 12, and 24 h postoperatively (all *p* < 0.01). Quadriceps strength was fully preserved in both groups at all assessed timepoints (*p* > 0.9999). **Conclusions**: The addition of perineural dexamethasone to a low-volume PENG block provides longer-lasting analgesia and reduces opioid requirements without compromising pain control or quadriceps function. This approach may improve the safety and efficacy of regional anesthesia in THA.

## 1. Introduction

Total hip arthroplasty (THA) is a major orthopedic procedure associated with substantial postoperative pain, which may hinder early mobilization and contribute to prolonged recovery, especially in older adults [1]. Effective analgesia is therefore a critical component of perioperative care and enhanced recovery pathways [2]. Although systemic opioids remain widely used, their adverse-effect profile—including respiratory depression, delirium, constipation, urinary retention, and increased fall risk—is particularly problematic in geriatric populations. Consequently, current practice increasingly emphasizes motor-sparing regional anesthesia techniques to reduce opioid requirements while facilitating early ambulation [3].

The pericapsular nerve group (PENG) block has emerged as a promising motor-sparing technique for hip analgesia by targeting the articular branches of the femoral, obturator, and accessory obturator nerves [4]. However, its clinical performance remains closely related to the volume of local anesthetic used. Standard PENG protocols often employ volumes of 20 mL or more to achieve sufficient spread within the complex fascial anatomy of the anterior pelvis. Such volumes risk unintended spread to the femoral nerve and may induce quadriceps weakness, undermining early rehabilitation [5].

Reducing local anesthetic volume is a potential strategy to avoid motor impairment, but lower volumes may provide insufficient coverage of the anterior hip capsule and shorten analgesic duration [6]. Thus, it remains unclear whether a lower-volume PENG block can provide analgesia equivalent to standard-volume injections without compromising efficacy.

Dexamethasone is commonly used as an adjuvant in peripheral nerve blocks, but its ability to prolong local anesthetic duration is variable and technique-dependent, with mixed findings reported across studies [7]. Some evidence suggests potential prolongation through anti-inflammatory and membrane-stabilizing effects, whereas other studies demonstrate only modest or inconsistent benefit [8,9]. Importantly, it remains unclear whether dexamethasone can offset the shortened duration typically associated with reduced local anesthetic volumes in the PENG block. No prior study has systematically evaluated this interaction, and the relative contributions of adjuvant pharmacology versus volume-dependent spread remain uncertain. Importantly, the existing literature does not clarify whether prolonged analgesia in this setting results from the pharmacologic effect of dexamethasone, the local anesthetic volume, or their interaction [8].

Therefore, we designed a randomized, double-blind controlled trial to compare a standard-volume PENG block (20 mL of 0.2% ropivacaine) with a low-volume PENG block (10 mL of 0.2% ropivacaine plus 4 mg of perineural dexamethasone). Our primary objective was to determine whether the addition of dexamethasone enables a lower local anesthetic volume to achieve prolonged analgesia without impairing motor function. Secondary objectives included evaluating differences in opioid consumption, pain scores, quadriceps strength, inflammatory response, and glycemic effects. This study aims to address the current knowledge gap regarding volume reduction strategies for the PENG block and their interaction with commonly used adjuvants.

## 2. Methods

### 2.1. Study Design and Ethical Oversight

This investigator-initiated, double-blind, parallel-group, superiority trial was conducted at the Orthopedic Clinical Hospital affiliated with the Poznan University of Medical Sciences (Poland). The study protocol was reviewed and approved by the Bioethics Committee at the Poznan University of Medical Sciences, chaired by Prof. Maciej Krawczyński, under approval number 657/24, issued on 6 November 2024. All procedures were performed in accordance with the ethical standards of the institutional and national research committees, as well as the Declaration of Helsinki. The trial was prospectively registered on ClinicalTrials.gov (NCT06470334). Written informed consent was obtained from all participants before enrollment.

Patients were eligible for inclusion if they were aged 65 years or older and under 100 years, scheduled for elective total hip arthroplasty, capable of providing written informed consent, and able to reliably communicate symptoms and responses to the research team. Exclusion criteria included any condition that prevented first-person informed consent, such as cognitive impairment or significant language barriers that interfered with communication or comprehension. Patients who did not meet these criteria were excluded from the study before randomization. This approach ensured that all enrolled participants were able to understand and reliably use the NPRS for postoperative pain assessment.

### 2.2. Randomization and Blinding

Participants were randomized in a 1:1 ratio to receive either a standard-volume PENG block with 20 mL of 0.2% ropivacaine (PENG group) or a low-volume PENG block consisting of 10 mL of 0.2% ropivacaine with 4 mg of dexamethasone (PENG + DEX group). The randomization sequence was generated in advance using a computer-based block randomization algorithm (nQuery Advisor, Statistical Solutions, Boston, MA, USA), ensuring equal allocation across groups while minimizing allocation bias.

To maintain allocation concealment, assignments were placed in sequentially numbered, opaque, tamper-proof envelopes by an independent investigator not otherwise involved in the study. These envelopes were stored securely and opened only in the preoperative area immediately before intervention.

Blinding was rigorously maintained through role separation. Upon arrival to the operating room, the envelope corresponding to the patient’s study number was opened by the designated “intervention anesthesiologist”—a consultant anesthesiologist with no further involvement in perioperative care or outcome assessment. This anesthesiologist prepared and administered the randomized PENG block according to group allocation. Immediately after performing the block, the interventionist left the operating suite. It was replaced by a second anesthesiologist (“blinded anesthesiologist”), who managed all intraoperative anesthesia care and remained blinded to the intervention.

All other clinical and research personnel—including the surgical team, ward nurses, physiotherapists, pain management staff, and data collectors—were blinded to the group assignments. Patients were unaware of which volume or adjuvant they received, preserving double-blind conditions throughout the study.

Outcome assessments, including pain scores, opioid use, quadriceps strength, and laboratory markers, were conducted by two independent evaluators blinded to group allocation. Group identity was masked in the database until after the final data analysis, at which point unblinding was performed solely to interpret and report results. This protocol ensured a robust Level of blinding and minimized bias at all stages of data collection and interpretation.

### 2.3. Intervention

All enrolled patients received standardized spinal anesthesia in preparation for total hip arthroplasty. The procedure was performed at the L 3/L 4 interspace using a 27-gauge, 90 mm Sprotte needle (PAJUNK^®^, Geisingen, Germany). Patients were placed in the lateral decubitus position, and upon confirmation of cerebrospinal fluid flow, 4 mL of 0.5% ropivacaine was administered intrathecally to ensure adequate surgical anesthesia. After spinal anesthesia and prior to the surgical incision, patients were repositioned supine for the administration of the Pericapsular Nerve Group (PENG) block.

The PENG block was executed under ultrasound guidance using a curvilinear low-frequency transducer (4–8 MHz) with sterile coupling gel. The ultrasound probe was positioned in the transverse plane over the anterior inferior iliac spine (AIIS) and iliopubic eminence (IPE), with orientation adjusted to enhance visualization of relevant anatomical landmarks, including the iliopsoas muscle and tendon. A 22-gauge, 80 mm echogenic needle (Stimuplex Ultra 360^®^, B. Braun, Melsungen, Germany) was inserted in-plane using a lateromedial approach. The needle was carefully advanced under continuous visualization to a spot immediately lateral to the iliopsoas tendon, situated between the AIIS and the IPE.

After hydro-dissection with 0.5 mL of normal saline to confirm proper needle placement and ensure separation of fascial planes, the appropriate study-specific injectate was administered slowly and incrementally with repeated negative aspirations. Patients in the PENG group received 20 mL of 0.2% ropivacaine, whereas those in the PENG + DEX group received 10 mL of 0.2% ropivacaine combined with 4 mg of preservative-free dexamethasone, diluted to a final volume of 12 mL with 0.9% sodium chloride. The use of 0.2% ropivacaine is consistent with prior evidence demonstrating that this concentration provides selective sensory block with minimal quadriceps involvement, thereby supporting early mobilization. Care was taken in both groups to ensure the injectate remained confined laterally to the iliopsoas tendon to minimize the risk of femoral nerve involvement, which could potentially impair quadriceps motor function.

Throughout the procedure, the block was conducted under strict aseptic conditions, and the surgical team did not perform supplemental periarticular local infiltration at any point during or after the surgery. Both anesthesiologists performing the blocks had over five years of clinical experience in ultrasound-guided regional anesthesia and had completed specific procedural standardization training before the study.

### 2.4. Surgery and Perioperative Management

All total hip arthroplasties in this study were performed using the Direct Superior Approach (DSA). This minimally invasive, muscle-sparing technique preserves the short external rotators and posterior-capsule, facilitating early postoperative mobilization and minimizing the risk of dislocation. Surgical procedures were conducted by a consistent, experienced orthopedic team consisting of four board-certified orthopedic surgeons, each of whom had performed over 200 THA procedures before the study.

Each patient received a cementless hip prosthesis system comprising the Smith & Nephew Polarstem^®^ femoral stem and R 3^®^ acetabular cup, selected based on preoperative imaging and bone quality assessments. Standardized intraoperative protocols were maintained, including skin antisepsis with chlorhexidine–alcohol, draping, and the use of perioperative intravenous cefazolin (2 g), administered within 30 min prior to incision for surgical site infection prophylaxis.

Intraoperatively, sedation was maintained using a target-controlled infusion (TCI) of propofol, titrated within a range of 2–5 mg/kg/h to achieve a Ramsay Sedation Score of 2–3. The propofol rate was carefully adjusted based on the patient’ s hemodynamic and respiratory responses to ensure comfort while preserving spontaneous ventilation. Supplemental oxygen was delivered via a face mask at a flow rate of 3–5 L per minute, titrated to maintain peripheral oxygen saturation (SpO_2_) above 94%.

Throughout the surgery, patients were continuously monitored using standard ASA monitors, including non-invasive blood pressure, three-lead ECG, pulse oximetry, capnography (in selected cases), and temperature monitoring. Intraoperative fluid management adhered to goal- directed therapy principles, and all patients received balanced crystalloid solutions.

To minimize the risk of postoperative thromboembolic events, pharmacologic thromboprophylaxis was initiated with 40 mg of enoxaparin subcutaneously, starting 12 h after the procedure and continued once daily for 28 consecutive days. Early mobilization was encouraged, and patients were assisted to ambulate with a walker under the supervision of a physiotherapist approximately 10–12 h after surgery, contingent upon stable vitals and preserved motor function.

### 2.5. Postoperative Pain Management

Postoperative analgesia followed a standardized multimodal pain control protocol tailored to geriatric patients, aiming to optimize pain relief while minimizing opioid exposure. The regimen included regular administration of:

Acetaminophen 1 g intravenously every 6 h, initiated within 1 h of surgery completion. Metamizole 1 g IV every 6 h, providing additional non-opioid analgesia with antipyretic and spasmolytic effects. Ibuprofen 600 mg IV every 8 h, administered unless contraindicated by renal function or gastrointestinal risk.

All non-opioid analgesics were continued for at least 48 h postoperatively unless clinically contraindicated. Rescue analgesia consisted of 5 mg intravenous oxycodone administered when pain intensity reached ≥4 on the Numerical Rating Scale (NRS). Pain was assessed by two independent blinded investigators at predefined time points (4, 8, 12 and 24 h postoperatively) and upon patient request. All NRS scores and the exact timing of rescue opioid administration were recorded in the electronic medical chart from the end of surgery.

All three medications were continued for at least 48 h postoperatively unless otherwise clinically indicated. Breakthrough (rescue) pain was managed using intravenous oxycodone, administered in 5 mg boluses when a patient reported pain intensity of ≥4 on the Numerical Rating Scale (NRS). Pain assessments were conducted by blinded clinical staff at prespecified intervals and upon patient request. Total opioid consumption over the 48-h postoperative period was meticulously recorded from electronic medical records and converted into morphine milliequivalents (mEQ) using a standardized conversion ratio, where 1 mg of oxycodone = 1.5 mg of morphine. This conversion facilitated consistent comparison with existing literature and enabled robust quantification of opioid-sparing effects between study groups. Patients were also routinely evaluated for non-pain-related side effects of analgesics, including nausea, vomiting, dizziness, and sedation, and appropriate supportive care was provided as needed. Rescue opioid administration was patient-driven. Opioids were administered upon patient request or when NPRS ≥ 4 during scheduled assessments, ensuring standardized and reproducible criteria for breakthrough pain

### 2.6. Outcome Measures

The *primary outcome* of the study was the *time to first rescue opioid analgesia*, defined as the number of hours elapsed between the conclusion of surgery and the first administration of opioid medication for postoperative pain rated as inadequately controlled. Time to first opioid was measured from the end of surgery until the moment of first rescue oxycodone administration. This was operationalized as a Numerical Rating Scale (NRS) score of 4 or higher, which triggered the administration of a 5 mg intravenous bolus of oxycodone. The time to first rescue opioid dose was measured continuously over a 48-h postoperative observation window. The exact time point was recorded by ward personnel and confirmed by independent study investigators who were blinded to the patient’s treatment group.

*Secondary outcomes* included various variables designed to comprehensively evaluate postoperative analgesia, opioid consumption, neuromuscular integrity, and systemic inflammatory response. One key secondary endpoint was the total amount of opioid consumed within the first 48 h following surgery, recorded in milligrams of intravenous oxycodone. To standardize and facilitate comparison with other trials, the recorded doses were converted to morphine milliequivalents (mEQ) using the established ratio of 1 mg oxycodone to 1.5 mg morphine. These data were extracted from electronic medication records and verified by two blinded researchers.

Postoperative pain intensity was assessed using the 11-point Numerical Rating Scale (NRS), where 0 represented “no pain” and 10 denoted “the worst imaginable pain.” Measurements were conducted at four predefined time intervals: 4, 8, 12, and 24 h after surgery. Each evaluation was performed by two independent, blinded physicians trained in standardized pain assessment procedures. Postoperative pain was assessed at standardized institutional time points (4, 8, 12, and 24 h), corresponding to routine monitoring and early mobilization protocols for hip arthroplasty. Discrepancies in reported scores were resolved through consensus at the time of documentation.

Another secondary outcome was the proportion of patients in each group who required any rescue opioid analgesia within the 48-h study period. This was categorized as a binary variable (yes/no), reflecting whether a patient ever reached the threshold of NRS ≥ 4 necessitating opioid intervention.

Functional motor assessment was conducted by evaluating quadriceps muscle strength, specifically focusing on knee extension and hip adduction, at 4, 8, 12, and 24 h postoperatively. Muscle strength was assessed using the Medical Research Council (MRC) scale, a validated five-point scale where a score of 5 indicated normal strength and 0 represented complete paralysis. Examinations were conducted independently by two blinded clinicians. Each assessment involved verbal commands and passive resistance testing, with the final score based on mutual agreement between evaluators.

The study also monitored potential nerve injury, assessed retrospectively based on neurologic documentation in the orthopedic ward records at discharge. Nerve injury was categorized using a 5-point classification: 0 indicated no injury; 1, minor sensory paresthesia; 2, complete sensory anesthesia; 3, motor deficit with or without sensory symptoms; and 4, complex regional pain syndrome. Abnormal findings were verified independently by two reviewers blinded to treatment allocation.

Additional secondary outcomes included laboratory parameters reflective of systemic inflammation and metabolic response. Blood glucose concentrations were measured at 12, 24, and 48 h after surgery from venous blood samples collected by nursing staff. Samples were processed in the hospital’s central laboratory, and results were reviewed by blinded study personnel. In parallel, two key inflammatory indices—the neutrophil-to-lymphocyte ratio (NLR) and the platelet-to-lymphocyte ratio (PLR)—were also determined at the same time points. These markers served as surrogate indicators of systemic inflammatory response and were used to assess whether the addition of dexamethasone modulated postoperative immune activation.

All outcome assessments, whether clinical or laboratory-based, were conducted in a manner that maintained the blinding of both participants and evaluators to the treatment group assignment throughout the entire study duration. Final outcome values were determined by consensus between the two designated assessors.

### 2.7. Sample Size Calculation

The sample size was determined based on the primary outcome: time to first rescue opioid analgesia (in hours). Preliminary pilot data (*n* = 10, not included in final analysis) indicated a mean time to first opioid administration of 9.1 ± 1.7 h in the standard PENG group and 15.0 ± 1.5 h in the PENG + DEX group, corresponding to a mean difference of 5.9 h. This difference was considered clinically meaningful, consistent with previous literature emphasizing opioid-sparing benchmarks in regional anesthesia.

Using these parameters, we applied a two-sample, two-sided *t*-test to estimate the required sample size, assuming α (type I error) = 0.05, β (type II error) = 0.05, and Power = 95%, with a pooled standard deviation of approximately 1.6 h (based on pilot data). Under these assumptions, a minimum of 5 patients per group (10 total) was required to detect a statistically significant difference in the primary endpoint.

To improve statistical robustness, detect clinically relevant secondary endpoints (e.g., opioid consumption), and accommodate for potential protocol deviations, dropouts, or incomplete data, the sample size was expanded to 30 patients per group (60 total). This design also allowed for balanced block randomization and increased external validity without compromising the power of the primary endpoint.

Sample size calculations were conducted using nQuery Advisor version 8.7 (Statistical Solutions, Boston, MA, USA), a validated software for clinical trial design.

### 2.8. Statistical Analysis

All statistical analyses were performed using GraphPad Prism version 10.1.1 (GraphPad Software Inc., San Diego, CA, USA). The analysis protocol was prospectively defined and applied consistently to all endpoints in the study. The distribution of continuous variables was assessed for normality using the Shapiro–Wilk test. Variables that followed a normal distribution are reported as mean ± standard deviation (SD), whereas non-normally distributed variables are presented as median and interquartile range [IQR].

For comparisons between the two study groups, continuous outcomes, including time to first rescue analgesia, Numerical Rating Scale (NRS) scores, total opioid consumption, blood glucose concentrations, and inflammatory markers, were analyzed using the unpaired two-tailed *t*-test for parametric data or the Mann–Whitney U test for non-parametric data.

Categorical variables, including the proportion of patients requiring rescue opioids, ASA classification, and incidence of nerve injury, were evaluated using the Chi-square test or Fisher’s exact test, depending on the expected frequency in contingency tables. Ordinal outcomes such as muscle strength scores were assessed using the Kruskal–Wallis test when appropriate.

All statistical tests were two-sided, and a *p*-value of less than 0.05 was considered statistically significant. For each statistically significant result, 95% confidence intervals (CI) were calculated to quantify effect sizes and assess the precision of estimates. To further strengthen the analysis of the primary endpoint (time to first rescue opioid administration), the use of Kaplan–Meier survival analysis with log-rank testing is recommended for future iterations of this study, particularly to account for censored observations.

Throughout the data collection and analysis phases, group allocation remained blinded. Unblinding occurred only after final data entry, verification, and cleaning were completed to ensure integrity and to minimize analytical bias.

## 3. Results

### 3.1. Participant Flow

Seventy-two patients scheduled for elective total hip arthroplasty were initially assessed for eligibility between 7 November 2024, and 31 January 2025, at the Orthopedic Hospital of the Poznan University of Medical Sciences. Of these, 8 patients were excluded before randomization. Specifically, 3 patients did not meet the predefined inclusion criteria, while 5 declined to participate after being informed about the study’s objectives and procedures. Following screening, 64 patients were successfully enrolled and randomized in a 1:1 ratio using block randomization to one of two study arms: the PENG group (standard 20 mL ropivacaine block) and the PENG + DEX group (reduced volume ropivacaine with 4 mg dexamethasone).

In the PENG group, 32 patients were randomized. Of these, 30 received the allocated intervention. Two patients did not receive the assigned intervention: one was excluded due to a failed spinal anesthesia, and one developed intraoperative surgical complications prior to block placement, leading to a protocol deviation.

**Figure 1:**
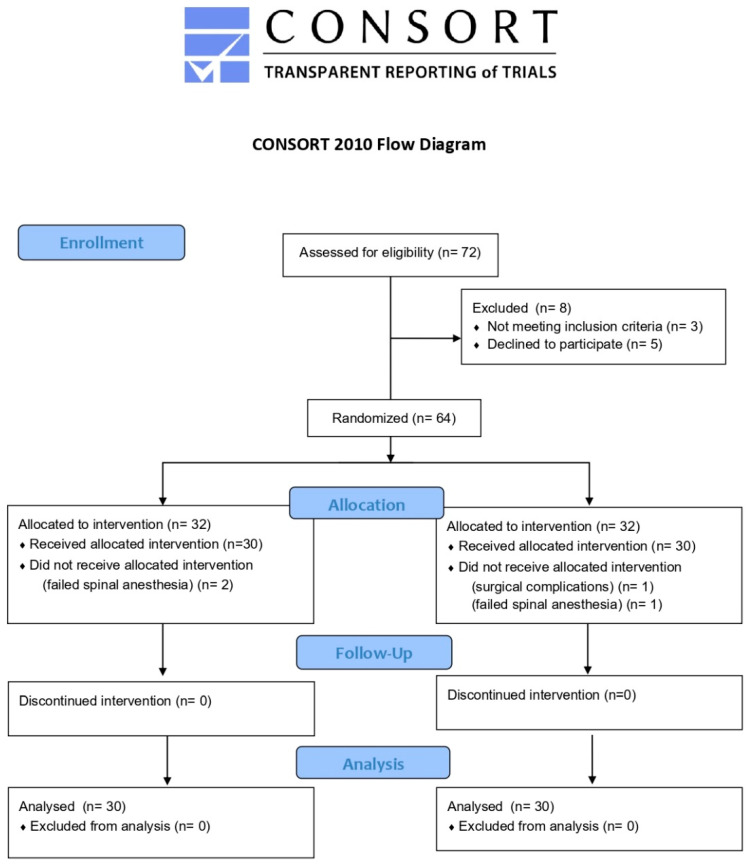
CONSORT 2010 Flow Diagram.

Similarly, in the PENG + DEX group, 32 patients were randomized. Again, 30 received the intervention as allocated, while two patients were excluded due to failed spinal anesthesia, which precluded continuation of the standardized anesthesia protocol. No participants in either group discontinued the intervention after administration, and no patients were lost to follow-up during the 48-h observation period. All patients who received the intervention were included in the final analysis. Thus, the final analysis was conducted on 60 patients: 30 in the PENG group and 30 in the PENG + DEX group, with no exclusions or missing data at the primary endpoint level, as seen in Figure 1. The target sample size of 60 was achieved. The additional four randomized patients were excluded before intervention due to protocol deviations and were not included in the analysis.

No clinically relevant differences were observed among the group characteristics, as shown in Table 1.

Flowchart depicting the enrollment, randomization, allocation, follow-up, and analysis of participants in the trial. A total of 72 patients were assessed for eligibility; 64 were randomized, and 60 completed the study protocol and were included in the final analysis.

### 3.2. Primary Outcome

**Figure 2:**
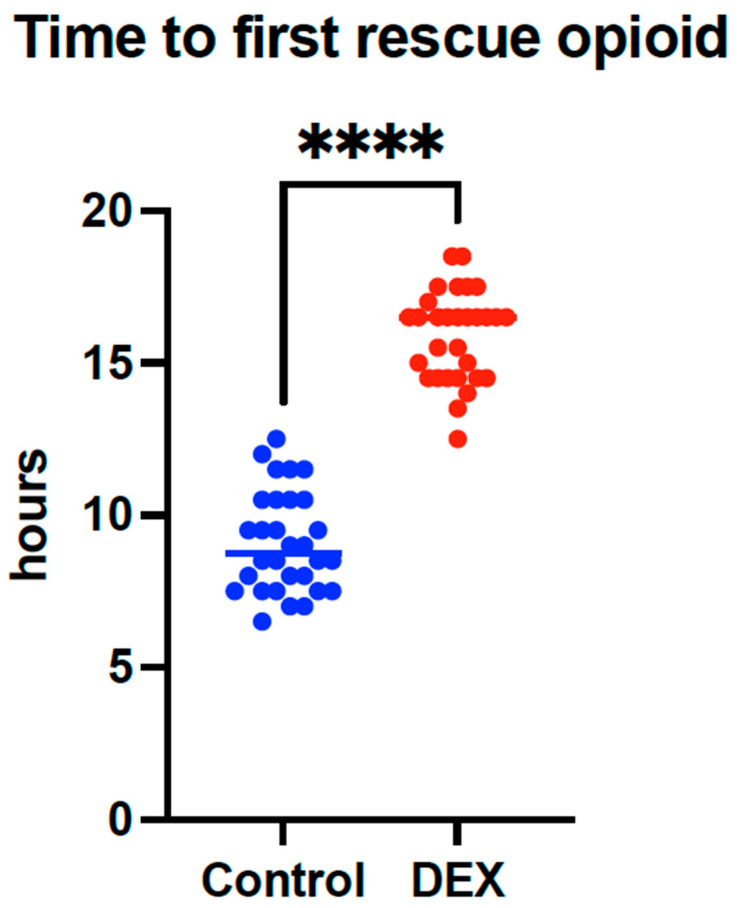
Time to First Rescue Opioid Administration (**** denotes *p* < 0.0001).

The primary outcome of the study, *time to first rescue opioid analgesia*, demonstrated a statistically and clinically significant difference between the study groups. Patients in the PENG + DEX group, who received 10 mL of 0.2% ropivacaine combined with 4 mg of dexamethasone, required their first opioid administration at a mean of 15.0 ± 1.5 h postoperatively. In contrast, patients in the standard PENG group, who received 20 mL of 0.2% ropivacaine, required opioids at a significantly earlier time point of 9.1 ± 1.7 h. This difference was highly significant, with a *p*-value of <0.0001 and a mean difference of 7.8 h (95% CI: 6.0 to 8.0), indicating a prolonged analgesic effect when dexamethasone was added to the block, as seen in Table 2 and Figure 2.

Comparison of primary (time to first opioid) and secondary outcomes (opioid consumption, pain scores, quadriceps strength, inflammatory markers, glucose levels) between groups. Values are shown as mean ± SD or number (%). Significant differences are highlighted.

Mean time (±SD) to first postoperative opioid administration in the PENG and PENG + DEX groups. The addition of dexamethasone significantly prolonged the time to rescue analgesia (*p* < 0.0001).

### 3.3. Secondary Outcomes

*Total opioid consumption* within the first 48 h after surgery was also significantly reduced in the PENG + DEX group. The mean opioid use, converted into morphine milliequivalents (mEQ), was 2.3 ± 3.1 in the PENG + DEX group compared to 5.0 ± 4.4 in the standard PENG group. This reduction reached statistical significance with a *p*-value of 0.0120. Furthermore, the proportion of patients who required any postoperative opioid rescue therapy was lower in the PENG + DEX group (12 patients, 40%) than in the PENG group (21 patients, 70%), and this difference was statistically significant (*p* = 0.0370), supporting the opioid-sparing effect of the dexamethasone-enhanced block.

*Pain scores* assessed using the Numerical Rating Scale (NRS) at multiple postoperative time points revealed comparable values between the groups at 4 h after surgery, with both groups reporting a mean score of 1.5 ± 0.6 (*p* = 0.8936). However, starting from the 8-h time point, the PENG + DEX group reported significantly lower pain intensity. At 8 h, the mean NRS was 2.0 ± 1.2 in the PENG + DEX group compared to 3.1 ± 1.1 in the PENG group (*p* = 0.0005). This difference remained statistically significant at 12 h (1.7 ± 0.8 vs. 2.5 ± 1.1, *p* = 0.0023) and at 24 h (1.4 ± 0.5 vs. 1.9 ± 0.7, *p* = 0.0014), suggesting that the analgesic benefits of the adjuvant extended beyond the early postoperative period.

*Quadriceps muscle strength* was fully preserved in all patients throughout the 24-h observation period. Both groups exhibited consistent scores of 5.0, without any variation, at all assessment time points for both knee extension and hip adduction. Statistical analysis showed no difference between groups (*p* > 0.9999), confirming that neither the reduced volume of ropivacaine nor the addition of dexamethasone compromised motor function.

No cases of *nerve injury* were observed in either group. All participants were graded as 0 on the predefined nerve injury scale (0 to 4) at discharge, and no transient or persistent neurologic symptoms were documented. There was no statistically significant difference in nerve safety outcomes between groups (*p* > 0.9999).

*Systemic inflammatory responses* were assessed using the neutrophil-to-lymphocyte ratio (NLR) and the platelet-to-lymphocyte ratio (PLR), measured at 12, 24, and 48 h postoperatively. No significant differences were observed at any time point. At 12 h, NLR values were 2.0 ± 0.7 in the PENG group and 1.7 ± 0.5 in the PENG + DEX group (*p* = 0.0554). At 24 and 48 h, values remained statistically comparable. Similarly, PLR values at 24 h were 339.9 ± 73.3 in the PENG group and 368.3 ± 82.3 in the PENG + DEX group (*p* = 0.2430), with no significant intergroup variation at any other time points.

*Blood glucose* levels were monitored at 12, 24, and 48 h postoperatively to assess potential glycemic effects of dexamethasone. Mean glucose concentrations remained stable and within the normal range in both groups, with no statistically significant differences. At 24 h, the PENG group recorded a mean glucose level of 122.0 ± 19.3 mg/dL compared to 116.2 ± 16.5 mg/dL in the PENG + DEX group (*p* = 0.1906). These findings suggest that the addition of 4 mg dexamethasone did not result in clinically meaningful alterations in postoperative glucose metabolism.

No PONV events occurred in either group during PACU or ward stay. As no events were observed, formal statistical comparison was not performed. We speculate that the reduced need for postoperative opioids in the PENG + DEX group may also reduce expected PONV incidence.

Collectively, these results demonstrate that the use of dexamethasone as an adjuvant in PENG block significantly extends the duration of analgesia, reduces opioid requirements, and improves pain control without compromising motor function or inducing systemic complications.

## 4. Discussion

This randomized controlled trial evaluated whether adding perineural dexamethasone to a reduced-volume ropivacaine PENG block could preserve analgesic efficacy while minimizing motor blockade and systemic opioid consumption in patients undergoing total hip arthroplasty (THA) via the direct superior approach (DSA). The findings indicate that the addition of dexamethasone significantly prolongs the duration of postoperative analgesia and reduces opioid use, without compromising quadriceps motor function or inducing metabolic or inflammatory complications. The use of 0.2% ropivacaine is consistent with prior evidence demonstrating that this concentration provides selective sensory block with minimal quadriceps involvement, thereby supporting early mobilization [4].

The most notable outcome was the significant extension in time to first rescue opioid administration in the PENG + DEX group, suggesting that dexamethasone enhances the duration of the nerve block even when the volume of local anesthetic is halved. This finding aligns with previous literature demonstrating the efficacy of dexamethasone as a block-prolonging adjuvant in peripheral nerve blocks [4,10]. The reduced need for postoperative opioids also supports the role of dexamethasone in opioid-sparing strategies, a crucial consideration in elderly patients prone to opioid-related adverse events [11]. The absolute prolongation of the analgesic duration was 7.8 h (95% CI 6.0–8.0). In the context of hip arthroplasty, extending analgesia into the first postoperative night and early physiotherapy period is clinically meaningful for mobilization and enhanced recovery.

Although both groups experienced adequate analgesia, the PENG + DEX group reported significantly lower pain scores at several postoperative time points. However, while statistically significant, these differences were relatively modest. Existing literature suggests that the minimum clinically important difference (MCID) in pain scores after THA is approximately 18.6 mm on a 100 mm visual analogue scale [12,13]. Therefore, the clinical relevance of the observed differences should be interpreted with caution and evaluated further in future studies designed with MCID thresholds in mind.

The study also highlights an important consideration regarding the selection of volume for PENG blocks. The comparison between 20 mL of plain ropivacaine and 10 mL of ropivacaine with dexamethasone demonstrates that the use of adjuvants may enable volume reduction without compromising analgesic efficacy [14]. Given that volumes of 10–15 mL are commonly used in clinical practice, future trials incorporating an intermediate control group (10 mL ropivacaine alone) would help isolate the independent effects of volume reduction versus pharmacologic enhancement [15]. Nonetheless, our findings suggest that a lower volume of local anesthetic may still be practical when appropriately augmented [16].

An additional consideration is the route of dexamethasone administration [17]. This study utilized perineural dexamethasone, although previous research has shown comparable efficacy between the perineural and systemic routes in specific settings [18]. We chose the perineural approach based on its potential for localized, prolonged action with minimal systemic exposure [8,9,19]. However, a head-to-head comparison of administration routes remains warranted to clarify whether perineural application provides any unique advantage.

The surgical approach also influences the interpretation of our results. All THA procedures were performed using the direct superior approach, which predominantly affects the posterior hip and sciatic nerve distribution [20]. Although the PENG block primarily targets the anterior capsule, our data showed satisfactory analgesia across both groups without the addition of a sciatic nerve block [21,22]. This finding suggests that the modified PENG technique used in this study may offer sufficient coverage when combined with a comprehensive multimodal analgesic regimen [2]. Nevertheless, further investigation is needed to determine whether additional blocks would enhance outcomes in posterior approach total hip arthroplasty.

Of note, the injection technique used in this study differed slightly from the original description of the PENG block [21], as the injectate was carefully confined lateral to the iliopsoas tendon to avoid femoral nerve spread. This modification, while aimed at preserving motor function, may restrict generalizability to standard PENG approaches [23]. Future studies would benefit from including ultrasound imaging to support reproducibility and precision in technique.

The metabolic safety of dexamethasone was also evaluated. No significant differences were observed between groups in blood glucose levels or systemic inflammatory markers, such as the neutrophil-to-lymphocyte and platelet–to–lymphocyte ratios. Dexamethasone was not associated with clinically relevant adverse effects, consistent with prior literature on low-dose perineural corticosteroids [8,19,24]. These findings support the short-term safety of a single perineural dexamethasone dose, consistent with earlier reports in orthopedic populations [25,26].

From a physiological perspective, a mild and transient perioperative hyperglycemia—when it occurs—is generally preferable to neuroglycopenia, even in patients with impaired glucose metabolism, and importantly no clinically relevant hyperglycemic episodes were observed in our cohort [27,28]. This is consistent with previous evidence demonstrating low incidence of clinically significant adverse effects with single-dose dexamethasone in regional anesthesia.

Hemodynamic safety and the potential for local anesthetic systemic toxicity (LAST) are important considerations when modifying local anesthetic dosing strategies. Although the PENG block is generally regarded as a low-risk technique, even relatively low ropivacaine doses have been implicated in hemodynamic instability under certain conditions. Stasiowski et al. [29] demonstrated that small changes in local anesthetic dosing during combined general–epidural anesthesia can precipitate clinically significant hemodynamic fluctuations. Moreover, rare but severe adverse events, including cardiac arrest associated with low-dose ropivacaine during femoral nerve blockade, have been reported in the literature [30]. These observations underscore the importance of careful dose selection, slow incremental injection, and consistent ultrasound visualization when performing fascial plane blocks in elderly patients.

Notably, no episodes of hemodynamic instability, LAST, arrhythmia, or cardiac arrest occurred in either group in the present trial, and no patient demonstrated early or delayed symptoms suggestive of systemic toxicity. All blocks were performed under real-time ultrasound guidance by experienced anesthesiologists, with incremental aspiration and standardized monitoring protocols. The absence of adverse physiological events in this cohort supports the methodological rigor and safety of both the standard-volume and reduced-volume ropivacaine regimens used in this study.

The findings of the present study are consistent with broader evidence demonstrating the potential of perineural dexamethasone to enhance postoperative analgesia across multiple regional anesthesia techniques [17,31,32]. In lower-limb surgery, dexamethasone has been shown to prolong sensory block duration and reduce opioid requirements when added to femoral nerve block, fascia iliaca block, and adductor canal block, without compromising motor function—an effect particularly relevant to early mobilization protocols in orthopedic populations [33,34,35]. Similar benefits have been observed with interfascial plane blocks, including the erector spinae plane block and transversus abdominis plane block, in which dexamethasone extends analgesic duration and improves early recovery profiles [19,31,36]. These observations support the hypothesis that dexamethasone may enhance the clinical utility of motor-sparing blocks such as the PENG block. However, the relative contribution of adjuvant pharmacology versus local anesthetic volume remains an area for future investigation.

Several limitations of this study must be acknowledged. The principal limitation of this trial is that it included only two treatment arms: 20 mL of 0.2% ropivacaine alone and 10 mL of 0.2% ropivacaine with dexamethasone. The absence of two additional comparison groups—10 mL ropivacaine alone and 20 mL ropivacaine with dexamethasone—prevents full separation of the effects of volume reduction from the pharmacologic effect of dexamethasone. Consequently, it remains unclear whether the observed differences reflect the impact of local anesthetic volume, the adjuvant, or their interaction. A future four-arm trial would be required to address this question definitively. Second, the follow-up period was limited to 48 h, which precluded the evaluation of long-term pain trajectories or functional outcomes. Third, although the study was appropriately powered for the primary endpoint, a larger sample size could have further strengthened the precision of secondary outcome analyses. Fourth, the single-center design and relatively homogeneous patient population may limit external generalizability.

In summary, this study demonstrates that the addition of 4 mg of perineural dexamethasone reduces ropivacaine volume by 50% in PENG blocks without compromising analgesic efficacy or motor function. Lastly, PONV was monitored throughout the postoperative period; however, no clinically relevant episodes occurred in either group, and Apfel score stratification was not part of the original protocol; therefore, PONV was not analyzed as a comparative endpoint. Given that the PENG + DEX group had significantly reduced postoperative opioid requirements, future studies should investigate whether reduced opioid exposure in this setting translates into lower PONV incidence and identify patients who might benefit from tailored antiemetic prophylaxis. This approach offers a safe and effective strategy for reducing systemic opioid requirements in patients undergoing THA, with potential advantages in enhanced recovery protocols focused on early mobilization and functional recovery. Future multicenter trials incorporating longer follow-up and comparative adjuvant strategies are warranted to validate and expand upon these findings.

## 5. Conclusions

In this randomized controlled trial, the addition of 4 mg of perineural dexamethasone to a reduced-volume PENG block significantly prolonged postoperative analgesia and decreased opioid requirements in patients undergoing total hip arthroplasty via the direct superior approach. The use of dexamethasone allowed for a 50% reduction in ropivacaine volume without compromising pain control or motor function, offering a clinically relevant strategy for motor-sparing, opioid-sparing regional anesthesia. Importantly, no metabolic or inflammatory adverse effects were observed, supporting the safety of perineural dexamethasone in this setting. These findings suggest that low-volume, adjuvant-enhanced PENG blocks may play a valuable role in enhanced recovery pathways, particularly for elderly patients undergoing hip surgery. Future multicenter trials with extended follow-up and comparative adjuvant arms are warranted to confirm these results and assess their impact on long-term functional outcomes and patient satisfaction.

## Figures and Tables

**Table 1:** Baseline characteristics.

	PENG	PENG + DEX
ASA		
ASA II	9	7
ASA III	21	23
Age (years)	71.9 ± 3.2 (66.0–78.0)	72.0 ± 3.1 (66.0–78.0)
M/F	14/16	15/15
BMI	29.2 ± 2.8 (25.0–36.0)	29.8 ± 2.9 (25.0–37.0)
Time of surgery (min)	77.5 ± 8.2 (60.0–95.0)	77.2 ± 7.4 (65.0–90.0)

PENG—Pericapsular nerve group; DEX—Dexamethasone; BMI—Body Mass Index; M—male; F—female; min—minutes; ASA—American Society of Anesthesiologists;

**Table 2:** Primary and secondary outcomes.

	PENG	PENG + DEX	*p*	Mean Difference (95%Cl)
Time to first rescue opioid analgesia (h)	9.1 ± 1.7	15.0 ± 1.5	<0.0001	7.8 (6.0 to 8.0)
48 h opioid consumption (morphine mEQ)	5.0 ± 4.4	2.3 ± 3.1	0.0120	−5.0 (−5.0 to 0.0)
Postoperative opioid consumption yes no	21 (70%) 9 (30%)	12 (40%) 18 (60%)	0.0370	N/A
NRS				
4 h	1.5 ± 0.6	1.5 ± 0.6	0.8936	0.0 (0.0 to 0.0)
8 h	3.1 ± 1.1	2.0 ± 1.2	0.0005	−1.5 (−2.0 to 0.0)
12 h	2.5 ± 1.1	1.7 ± 0.8	0.0023	0.0 (−1.0 to 0.0)
24 h	1.9 ± 0.7	1.4 ± 0.5	0.0014	−1.0 (−1.0 to 0.0)
Quadriceps muscle strength				
Knee extension				
4 h	5.0 (0)	5.0 (0)	>0.9999	0 (0 to 0)
8 h	5.0 (0)	5.0 (0)	>0.9999	0 (0 to 0)
12 h	5.0 (0)	5.0 (0)	>0.9999	0 (0 to 0)
24 h	5.0 (0)	5.0 (0)	>0.9999	0 (0 to 0)
Hip adduction				
4 h	5.0 (0)	5.0 (0)	>0.9999	0 (0 to 0)
8 h	5.0 (0)	5.0 (0)	>0.9999	0 (0 to 0)
12 h	5.0 (0)	5.0 (0)	>0.9999	0 (0 to 0)
24 h	5.0 (0)	5.0 (0)	>0.9999	0 (0 to 0)
Nerve damage (0–4)	0 (0)	0 (0)	>0.9999	0 (0 to 0)
NLR				
12 h	2.0 ± 0.7	1.7 ± 0.5	0.0554	−0.4 (−0.7 to 0.0)
24 h	3.9 ± 0.7	4.0 ± 0.9	0.7381	−0.2 (−0.4 to 0.5)
48 h	2.9 ± 0.7	3.1 ± 0.9	0.2648	0.1 (−0.2 to 0.7)
PLR				
12 h	181.4 ± 45.0	187.9 ± 70.9	0.8862	−4.8 (−33.1 to 31.8)
24 h	339.9 ± 73.3	368.3 ± 82.3	0.2430	9.8 (−12.1 to 69.2)
48 h	242.7 ± 62.1	262.5 ± 84.8	0.3600	6.0 (−24.9 to 61.3)
Blood glucose				
12 h	111.3 ± 17.1	112.9 ± 15.8	0.6620	4.0 (−6.0 to 10.0)
24 h	122.0 ± 19.25	116.2 ± 16.5	0.1906	−8.5 (−16.0 to 4.0)
48 h	113.9 ± 12.7	116.4 ± 17.1	0.6779	1.0 (−8.0 to 10.0)

PENG—Pericapsular nerve group; DEX—Dexamethasone; mEQ—milliequivalents; h—hours. *p*-value compares the PENG group to the PENG + DEX group.

## Data Availability

The data presented in this study are available on reasonable request from the corresponding author. The data are not publicly available due to privacy restrictions.
